# Thought Field Therapy Compared to Cognitive Behavioral Therapy and Wait-List for Agoraphobia: A Randomized, Controlled Study with a 12-Month Follow-up

**DOI:** 10.3389/fpsyg.2017.01027

**Published:** 2017-06-20

**Authors:** Audun C. Irgens, Asle Hoffart, Tor E. Nysæter, Vegard Ø. Haaland, Finn-Magnus Borge, Are H. Pripp, Egil W. Martinsen, Toril Dammen

**Affiliations:** ^1^DPS Aust-Agder, Sørlandet HospitalArendal, Norway; ^2^Research Institute, Modum BadVikersund, Norway; ^3^Department of Psychology, University of OsloOslo, Norway; ^4^Department of Psychiatry, Sørlandet HospitalArendal, Norway; ^5^Department of Psychiatry, Sørlandet HospitalKristiansand, Norway; ^6^Oslo Centre of Biostatistics and Epidemiology, Research Support Services, Oslo University HospitalOslo, Norway; ^7^University of Oslo, Institute of Clinical Medicine, Oslo University Hospital, Division of Mental Health and AddictionOslo, Norway; ^8^Department of Behavioral Sciences in Medicine, Institute of Basic Medical Sciences, Faculty of Medicine, University of OsloOslo, Norway

**Keywords:** thought field therapy, cognitive behavioral therapy, agoraphobia, psychotherapy, anxiety, energy psychology

## Abstract

**Background:** Thought field therapy (TFT) is used for many psychiatric conditions, but its efficacy has not been sufficiently documented. Hence, there is a need for studies comparing TFT to well-established treatments. This study compares the efficacy of TFT and cognitive behavioral therapy (CBT) for patients with agoraphobia.

**Methods:** Seventy-two patients were randomized to CBT (*N* = 24), TFT (*N* = 24) or a wait-list condition (WLC) (*N* = 24) after a diagnostic procedure including the MINI PLUS that was performed before treatment or WLC. Following a 3 months waiting period, the WL patients were randomized to CBT (*n* = 12) or TFT (*n* = 12), and all patients were reassessed after treatment or waiting period and at 12 months follow-up. At first we compared the three groups CBT, TFT, and WL. After the post WL randomization, we compared CBT (*N* = 12 + 24 = 36) to TFT (*N* = 12 + 24 = 36), applying the pre-treatment scores as baseline for all patients. The primary outcome measure was a symptom score from the Anxiety Disorders Interview Scale that was performed by an interviewer blinded to the treatment condition. For statistical comparisons, we used the independent sample’s *t*-test, the Fisher’s exact test and the ANOVA and ANCOVA tests.

**Results:** Both CBT and TFT showed better results than the WLC (*p* < 0.001) at post-treatment. Post-treatment and at the 12-month follow-up, there were not significant differences between CBT and TFT (*p* = 0.33 and *p* = 0.90, respectively).

**Conclusion:** This paper reports the first study comparing TFT to CBT for any disorder. The study indicated that TFT may be an efficient treatment for patients with agoraphobia.

**Trial Registration:** https://clinicaltrials.gov/, identifier NCT00932919.

## Introduction

Anxiety disorders are common (Kringlen et al., 2001), although the number of therapists delivering documented effective treatments has been limited (Collins et al., 2011). It is therefore of value to test other therapies aimed at reducing the symptoms of anxiety disorders, particularly therapies that practitioners can learn quickly and easily. One such group of therapies are the so-called energy psychology therapies, primarily in the forms of TFT (Callahan and Trubo, 2001) or EFT (Craig, 2007). These therapies consist of imaginal exposure combined with acupressure, i.e., tapping at acupoints, and aim to reduce anxiety over the course of a few sessions (Callahan and Trubo, 2001), thus having the potential to reach many patients. These methods may also be more acceptable for patients who are reluctant to experience *in vivo* exposure to feared situations, as is required in CBTs (Hawton et al., 1989).

In an overview from 2012 on research in the field of energy psychology therapies (Feinstein, 2012), 18 RCTs were described, of which 15 demonstrated large ES on at least one clinical outcome for TFT or EFT. One study showed a moderate ES (*d* = 0.67), while in two studies the ES could not be calculated. More recently, Church published RCTs demonstrating remarkably promising results from a small number of sessions with EFT for trauma symptoms (Church et al., 2012; Church, 2014; Church and Brooks, 2014; Church and Palmer-Hoffman, 2014).

Of the 18 RCTs, only two addressed the use of TFT for anxiety disorders (Schoninger and Hartung, 2010; Irgens et al., 2012). Schoninger and Hartung (2010) demonstrated a highly significant reduction (ES = 1.52) in public speaking anxiety following TFT (*N* = 28) compared to a wait-list condition (WLC) (*N* = 20). Irgens et al. (2012) found a significantly larger symptom reduction in patients with various, often comorbid, anxiety disorders treated with TFT (*N* = 23) compared to the WLC (*N* = 22) on 50% of the outcome measures. In these studies, the improvements were sustained at 5 and 12 months, respectively.

To further extend previous findings, the purpose of the present study was to perform a trial in which TFT was applied for a prevalent and invalidating anxiety disorder such as agoraphobia (Kringlen et al., 2001; Grant et al., 2006), and compare it to CBT, which is a well-established and empirically supported treatment of choice for this condition (Butler et al., 2006; Norton and Price, 2007; National Guideline Clearing House, 2011).

Imaginal exposure is a common method used in the treatment of anxiety disorders and PTSD, either alone or in combination with other therapeutic techniques such as CBT (Foa and Kozak, 1986; Arntz et al., 2007).

Both TFT and EFT apply imaginal exposure combined with tapping on acupoints (Church and Feinstein, 2013). They have their offspring in the clinical contributions of the American psychologist Roger Callahan (Callahan and Trubo, 2001), who sought to enhance the effect of CBT by tapping on specific points on the body, called acupoints, known from ancient Chinese medicine (Hui et al., 2000). TFT was first used as a treatment for anxiety disorders and traumatic memories, and has subsequently been used for an increasing number of conditions (Andrade and Feinstein, 2003; Feinstein, 2012).

The therapy commences with imaginal exposure, with the therapist asking the patient to focus on the selected incident, and to make it as vivid as possible. The therapist can help the patient through questions in relation to what the patient sees, smells, who is there and what is the most frightening thought just now or at the time of the incident (Eia, 2012). The patient is asked to rate his/her feelings on a Subjective Units of Distress Scale (SUDS) (Wolpe, 1990) from 0 to 10, with 0 being without negative emotion and 10 being as bad as the patient can imagine (Callahan and Trubo, 2001).

It is preferable that the patients do the tapping themselves, while the therapist instructs them on where and how to tap, often by tapping on themselves on the same acupoints as the patients are instructed to use. The tapping is performed from five to 10 times on each acupoint, firmly but not too hard. The patient clearly feels the tapping, though it should not cause any pain or bruising. According to the actual type of anxiety or other problem, the therapist chooses an algorithm, i.e., a sequence of acupoints prescribed in the TFT manual. The patient is asked to bear in mind the emotion or traumatic memory evoked by the therapist’s question, while the patient him/herself taps the chosen acupoints in the prescribed sequence. Sometimes, the therapist does the tapping at the beginning of the treatment, as it may be difficult for patients to concentrate on both their thoughts and emotions, while at the same time being instructed as to the proper acupoint to tap on, particularly during a state of high arousal.

The acupoints are situated on the hands and fingers, as well as on the face and the chest. There are one or more tapping algorithms for various negative emotions, and in addition to the algorithms comes a procedure that includes eye movements, counting and humming while tapping an acupoint on the edge of the hand (Callahan and Trubo, 2001).

Several theories have been proposed as to how the energy psychology therapies may acquire beneficial effects for psychiatric conditions (Ruden, 2007; Feinstein, 2010). However, based on a lack of empirical evidence, we evaluated the theories as being speculative. In addition to what is obtained by imaginal exposure, possible beneficial effects may have the same neurobiological correlates as in EMDR (Hartung and Galvin, 2003), a form of therapy that has been more studied than TFT and EFT. In a comprehensive discussion on possible theoretical models of EMDR, Bergmann (2010) described these theories as being speculative (Bergmann, 2010).

Thought field therapy may contain procedures partly being similar to those based on emotional processing theory. When the patient is asked to think about the problem, thought, images, feelings, and memories associated with the problem are elicited. Thus, this procedure can be conceived as a form of imaginal exposure to the problem (Foa et al., 2007). Imaginal exposure is based on emotional processing theory (Foa and Kozak, 1986). According to this theory, emotional processing is facilitated when the patient is emotionally connected with the problem, but at the same time feels in control and is not overwhelmed with anxiety. The effect of acupressure may be understood as promoting this process. By the gentle tapping, the patient is anchored in the here-and-now, being reminded that he/she is now sitting in the room and that his/her experiences are only thoughts and not reality. Moreover, when the therapist performs or instructs the tapping it reinforces the experience of the therapist’s presence, which may support the feeling of being helped and thus staying in control. The experience that the therapist uses or instructs a specific and concrete procedure (tapping across prescribed acupoints on the body) with certainty promotes the belief that the procedure is safe and effective. Together, these three factors may stimulate the feeling of being in control and this helps the patient overcome his/her tendency to suppress feelings while thinking about his/her problem and rather engage emotionally with the problem. Thus, we propose that TFT may work by potentiating the effect of imagery exposure. In addition, acupressure may have some so far unknown physiological or neurological effects.

Because TFT and EFT are applied by numerous therapists, both with and without a professional health-care education (Schwarz, personal communication, December 23, 2013), we wanted to study the efficacy of TFT in a randomized clinical trial. In addition, we think it is especially important with RCTs in the field of energy psychology, comparing TFT to a well-established psychotherapy as CBT, because there are only few such studies yet in this field.

## Materials and Methods

**FIGURE 1:**
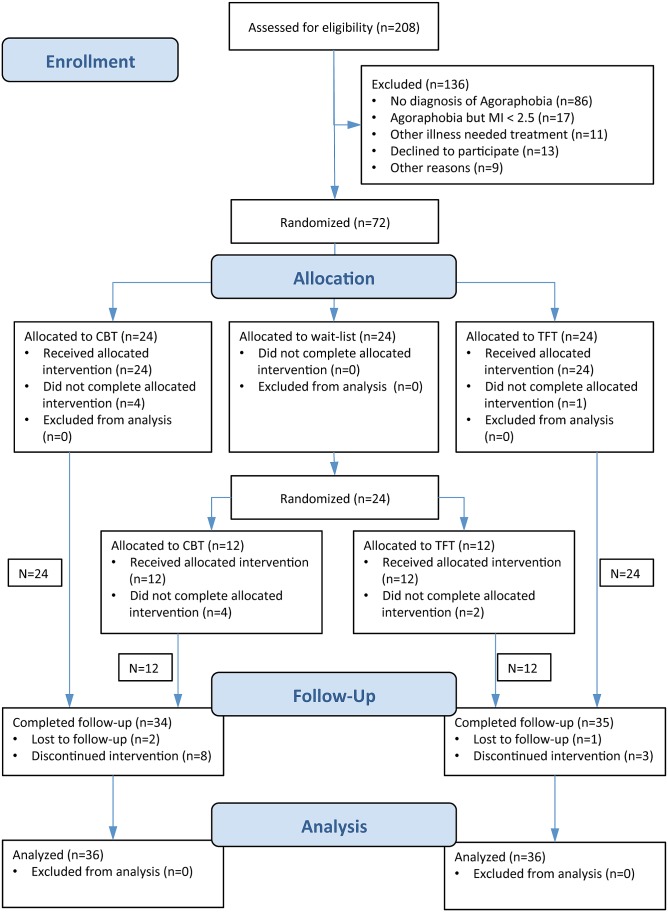
Flow diagram.

The study was designed as a longitudinal RCT comparing TFT (*N* = 24), CBT (*N* = 24) and a 3 months WLC (*N* = 24), **Figure 1**. We chose to include a wait list condition since TFT had not been tested for agoraphobia in previous studies. Three months duration of the WLC was chosen because the CBT treatment of 12 sessions was supposed to last for about 3 months.

### Recruitment

The patients were primarily recruited from the catchment area of the Sørlandet Hospital in the southern part of Norway between January 2007 and December 2008, after approval from the regional committee for medical and health research ethics. The leaders of the six outpatient clinics were informed both in writing and verbally about the study, and were asked to refer patients to the principal investigator (AI). Of the 72 patients, 51 (71%) were referred from these six outpatient clinics (*N* = 40, 57%) or from their GP (*N* = 11, 15%). News of the study was spread by word-of-mouth and through interviews in print media. As a result, 21 patients (29%) contacted the prime investigator directly (CBT *n* = 12, TFT *n* = 9).

Before diagnostic procedures were performed, patients were given verbal information about the study and gave their verbal consent to be screened. Patients were enrolled by the first author (AI), given a written description of the study and their written informed consent was also obtained after being assessed as being eligible for study inclusion. Treatment started in January 2007, and a FU was completed in September 2010.

### Study Entry Criteria

To be eligible, patients had to be 18 years of age or older and meet the DSM-IV criteria for the diagnosis of either “panic disorder with agoraphobia” or “agoraphobia without a history of panic disorder” as their principal disorder in need of treatment, as well as a high level of avoidance, measured as a score of 2.5 or higher on the MI-AAL (Chambless et al., 1985).

Exclusion criteria were a moderate to high risk of suicide, ongoing substance abuse or dependence, and a history of psychosis.

Patients were allowed to use any type of prescribed medication, although new medication for anxiety was not allowed to be initiated.

### Design

Seventy-two patients were randomly assigned to treatment with CBT (*N* = 24), TFT (*N* = 24), or a 3-month WL (*N* = 24), applying block randomization stratified by gender. The randomization was made by a statistician using the random number generator in SPSS, while the randomization key was kept secret from the study personnel by a secretary, and was only revealed to the principal investigator (AI) after the diagnostic procedures and blinded assessment were completed. After the waiting period, the WL patients (*n* = 24) were again randomly assigned by an identical randomization procedure as previously described to either CBT (*N* = 12) or TFT (*N* = 12).

### Assessments and Diagnostics

The ADIS interviews and self-report assessments (DiNardo et al., 1994) were conducted at pre-treatment, post-treatment, and at 12-month FU. Before inclusion, patients were assessed by the structured clinical interviews MINI PLUS (Sheehan et al., 1998) for DSM-IV Axis I and SCID II (American Psychiatric Association, 1994) for Axis II disorders, both of which were performed by the principal investigator (AI). Decisions on enrollment in the study were made by the principal investigator. Using video, 10 of the MINI PLUS and 10 of the SCID II interviews were reassessed by an experienced research psychiatrist (TD). There was a total agreement regarding the diagnoses of agoraphobia and panic disorder, and a consensus was reached for the presence of the other diagnoses after discussion. The MINI PLUS and SCID II interviews were repeated at the 12-month FU, and the interviewer (AI) knew the treatment condition of the patient at the time of the last assessment.

### Measures

The primary outcome measures were cumulative scores on avoidance and anxiety from the ADIS (DiNardo et al., 1994) for agoraphobia, as used by Craske et al. (2003). Secondary outcomes were measured using the following instruments: inference and distress from the ADIS, MI-AAL (Chambless et al., 1985), the BDI (Beck et al., 1961), the BAI (Beck et al., 1988), the ACQ, and the BSQ (Chambless et al., 1984).

The ADIS interviews (DiNardo et al., 1994) were performed by one of two psychologists or a psychiatric registrar, all of whom were blinded to the patient’s treatment condition. Before the start of the study, they scored three ADIS interviews from videos, and compared and discussed their performance with the first author (AI). All ADIS interviews were videotaped and reevaluated by the first author (AI), with feedback given to the interviewer when necessary.

To assess the competence of the CBT therapists, the supervisors scored 15 of the 20 supervised sessions using the CTS (Young and Beck, 1980, unpublished; Westra et al., 2011). The CTS comprises 16 items rated on a seven-point Likert scale from 0 (non-adherence to aspects of therapy) to 6 (adherence and very high skill). Item 15 is scored from 0 to 4. Items 1–6 characterized general therapeutic skills, and items 7–11 scored conceptualization, strategy and technique. Item 14 gave an overall rating, item 15 assessed whether you would select this therapist to participate if you were conducting an outcome study of cognitive therapy for agoraphobia, assuming the session was typical, with item 16 denoting how difficult the patient was to work with. For TFT no such competence scale exists.

At the 12-month FU, the patients were asked for possible side effects of treatments. This was posed as an open question, as we were not aware of what type of side effects to expect. The ADIS interviewers were asked to register at the 12 months FU whether the patients let them know which therapeutic option they had received, and to guess the treatment condition of each patient.

### Interventions

#### Thought Field Therapy

In this study, 36 patients were given a TFT treatment package of five 50- to 55-min sessions of individual therapy, which was standard procedure in Norway (Irgens et al., 2012). The treatment was based on the TFT guidelines created by Callahan and Trubo (2001), and as with most psychotherapy, the treatment started with taking the history and creating an overview of the patient’s social situation and risk factors. The TFT therapist asked for the first anxiety attack, traumatic incidents, the worst attack of anxiety and often also the most recent situation in which anxiety affected the patient. During therapy, patients were asked to: (a) recall the symptom that they wanted to get rid of, (b) report the present SUD score (Wolpe, 1990), and (c) focus on different aspects of the symptom while tapping on a sequence of acupressure points that the TFT therapist prescribed (Callahan and Trubo, 2001). Imaginal exposure was used to bring forth some of the feelings or bodily sensations of an anxiety experience. Most of the time and focus in the treatment were on feelings or bodily sensations. The SUD level was used during the sessions on many feelings or bodily sensations. Typically one bodily feeling was pressure in the chest area. With focus on this feeling the SUD could start at 7, when tapping it was reduced to 5. Then the therapist continued with the same algorithm and hopefully got a new reduction to 3 or lower. Often when the pressure in the chest was relieved a new feeling was noticed by the client, “it feels like a lump in the gut.” If no SUD reduction was achieved after following the procedure a question to identify a new or extended focus regarding the problem/feeling or situation in focus was applied. Common questions were: “What is the worst thing about that feeling? What is the worst that can happen in that situation?” Then the suitable algorithm was applied to treat the problem/feeling that was reported by the patient.

Often the therapist had to instruct the patients to use different algorithms to treat more complex problems arising during the therapy. If the patient thought of the problem from different “directions,” more complex problems could be solved by treating traumatic memories and other disturbing emotional reactions, aspect by aspect, in addition to the specific agoraphobic symptoms. With use of only the TFT algorithms for solving complex problems there is a need for more than one session, and in Norway the guideline has been five sessions as taught in the TFT education seminars by Uldal (2007; Holmaas, 2017). Most patients have complex problems and need more than one session to “unlock” several aspects of the patient’s difficulties. Using the algorithms we expected that the patient used the method by themselves. To secure that they learned properly how to do the tapping it was desirable with more than one session.

#### TFT Therapist and Training

An experienced TFT therapist, with no formal health education or therapeutic experience other than that of being a TFT therapist, conducted the TFT. In particular, she had no experience with CBT. The TFT therapist was certified in Norway to the Algorithm level in 2003 and to the TFT Diagnostic Level by Callahan Techniques Ltd. in 2005. Based on Callahan’s manual for anxiety disorders and panic attacks, she constructed a TFT manual in Norwegian specific for agoraphobia (Eia, 2012). The manual describes in detail how the TFT therapist guides the patient to target the memories and other thoughts that trigger maximum anxiety, and prescribes the specific sequence of acupoints to be tapped by either the patient or therapist. This manual was discussed with both the study’s principal supervisor (AH) and the principal investigator (AI), and has been used as a course manual for TFT therapist training in Norway. It is not published broadly but available in Norwegian upon request. The principal investigator supervised the TFT therapist during the study to assure adherence to the manual, as well as to ensure that she did evaluations of the risk of suicide and other issues on patient safety. Early- or middle-phase sessions with nine individual patients were videotaped and evaluated by another experienced TFT therapist familiar with the agoraphobia manual. The therapist who evaluated the TFT sessions was instructed to secure adherence to the TFT algorithms and to pay special attention to use of non-TFT approaches in the taped TFT sessions. Unfortunately, there is no standardized adherence measure available for TFT treatment that he could use.

#### Cognitive Behavioral Therapy

The CBT was conducted in accordance with a manual created by David M. Clark and Paul M. Salkovskis, a revised version of the manual described in Hawton et al. (1989). It consisted of 12 sessions of 50–55 min each. The first stage in therapy introduced and socialized the patient to the CBT model through collaboration between patient and therapist on creating an individualized cognitive model, based on the patient’s specific problems. The CBT model assumes that patients interpret bodily symptoms that normally occur during intense anxiety in a catastrophic way as signs of a bodily or mental disaster. An avoidance of triggers that elicit anxiety and safety behavior when confronted with triggers both serve to maintain the catastrophic cognitions. Following the model from Clark and Salkovskis, the CBT therapists aimed at forming a scientific team with their patients called “collaborative empiricism” (Beck, 1979), for using this therapeutic style to create experiments to test the meaning of the patients’ bodily symptoms. The most common was hyperventilation experiments and experiments to achieve a fast rise in pulse rates, where the patients’ interpretation of their symptoms was subsequently challenged to demonstrate that the fearful symptoms did not have dangerous causes, and that the symptoms would decline even if no action to reduce them was taken. Patient and therapist planned behavioral experiments to be performed between sessions, and in a few cases, some of the sessions were used to accompany the patient during exposure to out-of-office situations (e.g., elevators). In cognitive restructuring, such experiments aim to make the patients question the evidence supporting their catastrophic interpretation of the symptoms, and to help them strengthen alternative and more benign beliefs. In addition, the therapy aimed at encouraging the patients to explore that their need of avoidance and other safety strategies no longer existed, in order to let go of these anxiety maintaining behaviors.

#### CBT Therapists and Training

The two CBT therapists were experienced psychiatrists with formal training and certification in CBT. During the year before the start of the study, they were both trained in using the CBT manual for agoraphobia and panic disorders by two experienced cognitive therapists and researchers, who have a Ph.D. in CBT for agoraphobia (AH) and social phobia (FMB). During this period of training, the therapists were supervised biweekly with feedback by phone on the video recordings of therapy sessions. During the study, they each received feedback on 10 video recorded sessions.

### Data Analysis

The power calculations were originally performed for a non-inferiority study concept assuming 70% effectiveness for CBT and 85% for TFT, with 80% strength and 10% CI and an accepted 10% delta, yielding 34 patients in each therapy group. As both the criteria for estimating effectiveness for TFT, and the non-inferiority margin for scores on the ADIS avoidance scale are highly uncertain, we present our findings as a conventional superiority trial with two-sided statistical tests.

Statistics were performed first on the three conditions CBT 12 sessions (*n* = 24) and TFT five sessions (*n* = 24) after treatment and WL (*n* = 24) after a 3 months waiting period (**Table 3**), and on the two conditions CBT (*n* = 36) and TFT (*n* = 36) at 12-month FU, applying the pre-treatment scores as baseline for all patients (**Table 4**).

Independent sample’s *t*-test and Fisher’s exact test were used for a statistical comparison between the two groups for continuous and categorical data, respectively.

Repeated measures ANOVA was used to assess treatment across the three FU times, and ESs (Cohen’s *d*) were calculated using G^∗^Power 3^20^. To adjust for differences in pre-treatment symptom scores, ANCOVA analyses were applied with pre-treatment values for the primary effect measures as a covariate.

Because there were few missing data, only approximately 5%, we analyzed data using the principle of “intention to treat,” with the LOCF for the missing data.

### Ethics Approval and Consent to Participate

This study was carried out in accordance with the recommendations of the Regional committee for medical and health research with written informed consent from all subjects. All subjects gave written informed consent in accordance with the Declaration of Helsinki. The protocol was approved by the regional committee for medical and health research ethics (reference number S-06019). The study was registered in the clinicaltrials.gov in 2006, but by a mistake it was not registered as received until July 3rd, 2009.

## Results

Recruitment and participation are presented in the flowchart (**Figure 1**). Of the 208 patients assessed for eligibility, 72 were found eligible, consented to study participation and were randomized to CBT, TFT, or WL. There were no differences between the treatment groups in terms of receiving allocated treatment or participating at FU (**Figure 1**).

No significant statistical differences were found between the groups regarding patient characteristics at baseline (**Tables 1**, **2**). Seventy-one of the patients had panic disorder with agoraphobia, while one patient had agoraphobia without panic disorder. For the 136 patients that were excluded from the study we only have data for gender and age at assessment, which were not significantly different from the 72 study patients.

**Table 1:** Demographic data for the three original groups.

Characteristic	Group		
	
	TFT (*n* = 24)	WL (*n* = 24)	CBT (*n* = 24)
	*M* (*SD*)	*M* (*SD*)	*M* (*SD*)
Age at study start, years	39.3 (11.8)	40.9 (12.7)	33.8 (12.0)
Duration of symptoms, years	11.1 (8.5)	15.1 (13.4)	11.7 (9.5)
Number of Axis I diagnoses, mean	2.0 (1.2)	2.3 (1.6)	2.7 (1.3)

	***n* (%)**	***n* (%)**	***n* (%)**
	
Female	18 (75)	18 (75)	18 (75)
Affective disorder, current^∗^	4 (17)	7 (29)	10 (42)
Affective disorder, lifetime^∗^	14 (58)	15 (33)	18 (75)
One or more anxiety disorders, in addition to agoraphobia with/without panic disorder	9 (38)	11 (46)	11 (46)
Abuse of alcohol or drugs, lifetime	1 (4)	3 (13)	3 (13)
Number of patients diagnosed with one or more personality disorders	4 (17)	6 (25)	4 (17)
Number of patients regularly using benzodiazepines	2 (8)	5 (21)	1 (4)
Number of patients occasionally using benzodiazepines	3 (13)	6 (25)	5 (21)
Number of patients using antidepressants	14 (58)	15 (63)	12 (50)


*^∗^Includes major depressive episode, recurrent depression, bipolar disorder type 2 and dysthymia.*

**Table 2:** Demographic data for the two therapy groups.

Characteristic	Group	
	
	TFT (*n* = 36)	CBT (*n* = 36)
	*M* (*SD*)	*M* (*SD*)
Age at study start, years	39.1 (12.2)	36.9 (12.7)
Duration of symptoms, years	11.8 (8.8)	13.5 (12.2)
Number of Axis I diagnoses, mean	2.1 (1.4)	2.5 (1.4)

	***n* (%)**	***n* (%)**
	
Female	27 (75)	27 (75)
Affective disorder, current^∗^	8 (22)	13 (36)
Affective disorder, lifetime^∗^	21 (58)	26 (72)
One or more anxiety disorders, in addition to agoraphobia with/without panic disorder	15 (42)	16 (44)
Abuse of alcohol or drugs, lifetime	4 (11)	3 (8)
Number of patients diagnosed with one or more personality disorders	9 (25)	5 (14)
Number of patients regularly using benzodiazepines	4 (11)	4 (11)
Number of patients occasionally using benzodiazepines	6 (17)	8 (22)
Number of patients using antidepressants	21 (58)	20 (56)


*^∗^Includes major depressive episode, recurrent depression, bipolar disorder type 2 and dysthymia.*

Over the period of treatment and FU, the proportion of patients who changed their psychotropic medication (*n* = 27) did not significantly differ between patients receiving CBT (*n* = 15) or TFT (*n* = 12). Furthermore, there were no significant between-group differences among patients who reduced (TFT *n* = 8, CBT *n* = 10) or increased their dosage (TFT *n* = 4, CBT *n* = 5). Patients who used benzodiazepines (Bz.) regularly or occasionally showed a trend toward lesser reduction on the primary outcome of avoidance than the other patients in the study both pre-post (Bz. = 1.19, no Bz. = 1.79, *p* = 0.09) and pre-12 months FU (Bz. = 1.13, no Bz. = 1.64, *p* = 0.19), and no difference between CBT or TFT (*p* = 0.80).

The proportion of patients who received additional treatment during the trial (*n* = 14) did not significantly differ between the CBT- (*n* = 8) and the TFT (*n* = 6) condition. Additional treatments were supportive therapy (CBT = 2, TFT = 3), three-five extra sessions with their study therapist due to acute situations not related to their agoraphobia (CBT = 1, TFT = 2), other therapist contact (CBT = 4, TFT = 1) and acute inpatient care (CBT = 1).

Eleven patients (CBT = 8, 22%, TFT = 3, 8%, Fisher’s exact test, *p* = 0.19) dropped out of the scheduled treatment at various stages. In the CBT group, the dropouts completed 2–10 of the 12 scheduled sessions; the three TFT dropout patients completed 3–4 of the 5 sessions. Among the dropouts, three (CBT = 2, TFT = 1) ended their therapy because they experienced significant symptom reduction and experienced no need for further sessions, whereas one TFT patient dropped out due to moving to another part of the country, but experienced considerable symptom reduction after three sessions. The reduction in the primary effect variable of avoidance symptoms did not differ between dropouts from CBT (0.98, CI 1.64) or TFT (0.42, CI 0.49), *F* 0.09, *p* 0.77.

Three of the 11 dropouts were lost to FU (CBT = 2, TFT = 1), while eight attended the FU evaluations (CBT = 6, TFT = 2). Among these eight, three did not fill in self-report measures post-treatment, but did attend the interview for assessing the primary effect variable.

### Reliability Testing

Due to practical reasons, 47 of the 227 ADIS interviews were performed by the first author. When rescored from video by two of the primary interviewers (ES, VØH) who were blinded to the first author’s score, the intra-class correlation (ICC) was 0.94 (CI 0.90 – 0.97). These results compare well with those found by Brown et al. (2001), who reported a Pearson’s *r* of 0.86 for the rating of agoraphobic avoidance from the ADIS.

### Treatment Integrity

Seven CBT sessions from the starting phase and eight from the mid-phase were assessed using the CTS. For both items 1–6 and items 7–11, the therapists were scored as being good, with a mean of 4.0 (range 3.2–4.8) and 4.0 (range 2.6–5.0), respectively. For item 14 the mean was 3.9 (range 2.5–5.0), with a score of 4 describing the therapist as good. For item 15, the mean score was 3.0 (range 1.0–4.0), which means a likely “yes” to the question on choosing this therapist for a new study. For item 16, the mean was 1.4 (range 0.0–4.0), with a score of 0 saying that the patient was not difficult, while a 3 denotes a medium difficult patient.

There is no systematic treatment fidelity instrument developed for TFT. The external experienced TFT therapist, who viewed nine sessions by video, confirmed that the content of the TFT treatment was in accordance with standard content and procedures in all sessions.

### Primary Outcomes

**FIGURE 2:**
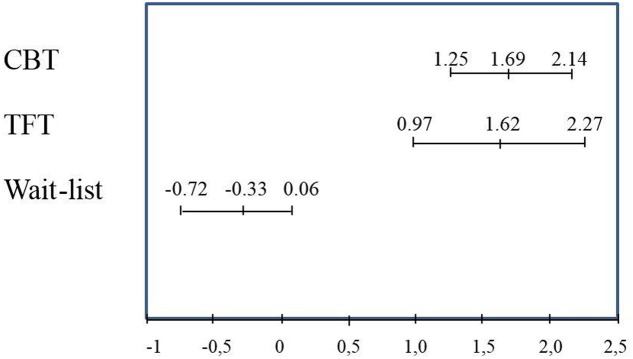
Means and confidence intervals for changes on the ADIS symptom score for avoidance, from before to after wait-list (*n* = 24) or treatment with CBT (*n* = 24) or TFT (*n* = 24).

**Figure 2** shows the comparison of changes between CBT, TFT, and the WL from before to after the end of therapy or the waiting period for the avoidance scale from the ADIS, *a priori* chosen as the primary effect variable. There were significant differences between the WL group and the two treatment groups, with *p* < 0.001 for the comparisons between WL patients and patients who received either CBT or TFT.

**FIGURE 3:**
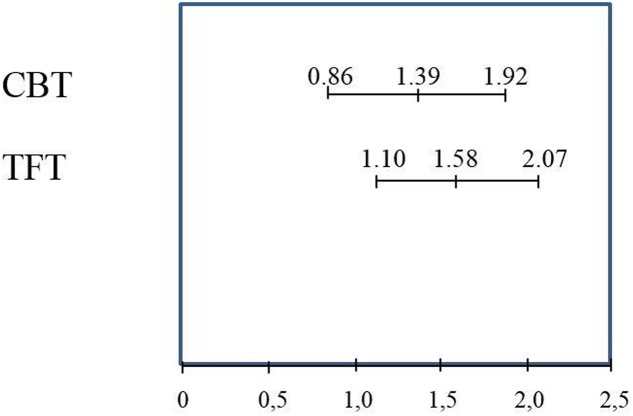
Means and confidence intervals for changes on the ADIS symptom score for avoidance, from before to 12 months after treatment with CBT (*n* = 36) or TFT (*n* = 36).

**Figure 3** shows the comparison of beneficial changes between CBT and TFT from before treatment to the 12-month FU for the avoidance scale from the ADIS.

**FIGURE 4:**
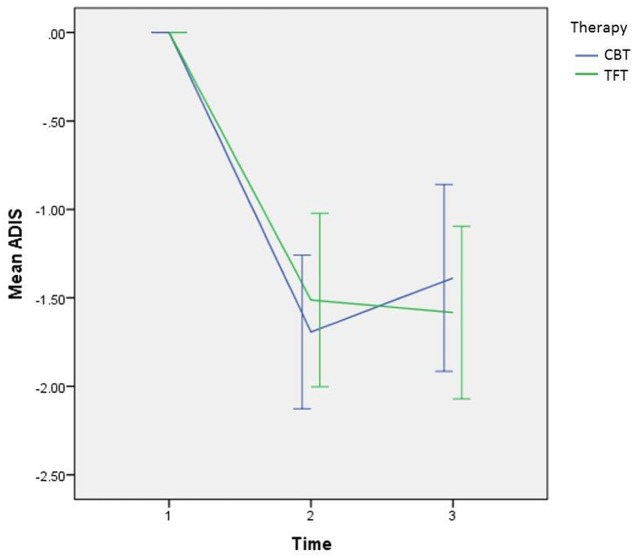
Curves showing mean changes with a 95% CI in the primary effect variable of avoidance from the ADIS, from before to after treatment with CBT (*n* = 36) or TFT (*n* = 36), and from before to 12 months after treatment.

**Figure 4** illustrates the mean changes of the primary effect variable across the three time points in the study.

The effects of the two assigned treatment modalities were assessed with an ANCOVA model, adjusting for the initial level of avoidance before treatment. For our primary outcome, the mean difference between the CBT and TFT groups after treatment was non-significant: -0.31 (95% CI -0.93 – 0.32), and *p* = 0.33 for the ADIS avoidance scale. The mean difference between the CBT and TFT groups on the 12-month FU was also non-significant: 0.041 (95% CI -0.63 – 0.72), and *p* = 0.90 for the ADIS avoidance scale.

### Secondary Outcomes

**Table 3** shows the comparisons of changes between CBT, TFT, and the WLC from pre to post therapy or waiting period for all parameters, including ESs, with *F*-values for CBT and TFT both computed in relation to the WL patients. There were significant differences between the WL group and the two treatment groups on all parameters, showing that CBT and TFT did better than WL (*p* < 0.05), except for BSQ for which the *p*-value was 0.050 for CBT and 0.052 for TFT. When comparing the CBT (*n* = 24) and TFT (*n* = 24) groups at baseline, no parameters showed significant *F*-values.

**Table 3:** Mean, standard deviations, and effect sizes (Cohen’s *d*) for patients randomized to treatment with (1) CBT 12 sessions (*n* = 24) or (2) TFT five sessions (*n* = 24), or (3) a 3 months wait-list (*n* = 24), measured at baseline and after treatment or waiting period. *F*-values were computed for CBT and TFT in relation to wait-list, as a Condition × Time effect across the assessments.

	(1) Immediately before treatment or waiting period		(2) Immediately after treatment or waiting period				
					
	*M*	*SD*	*M*	*SD*	*d*	*F*(1,48)	*P*
**ADIS anxiety**							
CBT	2.91	1.16	1.24	1.32	1.60	41.4	
TFT	3.57	1.44	1.95	1.67	1.11	25.2	0.89
Wait-list	3.13	1.02	3.28	1.30	-0.17		
**ADIS avoidance**							
CBT	2.70	1.23	1.01	1.31	1.61	49.7	
TFT	3.26	1.59	1.64	1.76	1.06	28.2	0.85
Wait-list	2.85	0.95	3.18	1.34	-0.39		
**ADIS inference**							
CBT	6.17	1.58	2.38	2.28	1.75	27.0	
TFT	5.67	1.93	3.21	2.52	1.02	7.38	0.06
Wait-list	6.33	1.01	5.54	1.56	0.47		
**ADIS distress**							
CBT	6.08	1.61	2.50	2.38	1.63	36.9	
TFT	6.08	1.74	3.54	2.40	1.02	14.8	0.14
Wait-list	6.17	1.40	5.92	1.61	0.18		
**MI alone**							
CBT	3.35	0.55	2.16	0.85	1.78	39.1	
TFT	3.56	0.66	2.62	1.00	0.89	11.7	0.35
Wait-list	3.42	0.58	3.30	0.64	0.29		
**BDI**							
CBT	16.3	9.68	11.1	9.35	0.90	8.26
TFT	13.8	8.28	8.71	7.23	0.55	4.81	0.94
Wait-list	14.1	11.8	14.3	12.5	-0.03		
**BAI**							
CBT	25.8	13.6	13.9	13.6	0.94	15.7
TFT	21.4	10.8	14.0	10.8	0.58	5.40	0.22
Wait-list	25.5	14.0	25.0	14.4	0.08		
**ACQ frequency**							
CBT	2.14	0.57	1.72	0.55	0.99	14.0
TFT	2.07	0.45	1.68	0.46	0.70	9.42	0.88
Wait-list	2.32	0.54	2.38	0.74	-0.15		
**ACQ belief**							
CBT	25.3	15.1	11.5	10.8	1.18	13.6
TFT	24.0	11.7	13.0	12.0	0.75	7.68	0.50
Wait-list	31.7	14.2	32.6	21.1	-0.07		
**BSQ**							
CBT	2.71	0.75	2.09	0.70	0.78	4.04
TFT	2.60	0.63	1.98	0.71	0.77	3.97	1.00
Wait-list	2.90	0.86	2.72	1.02	0.26		


**P*-values were computed for the differences between CBT and TFT.*

*CBT, cognitive behavioral therapy; TFT, thought field therapy; ADIS anxiety, mean symptom score on anxiety; ADIS avoidance, mean symptom score on avoidance; ADIS inference, inference on daily life from agoraphobic symptoms; ADIS distress, distress in daily life from agoraphobic symptoms; MI alone, Mobility Inventory, subscale alone; BDI, Beck Depression Inventory; BAI, Beck Anxiety Inventory; ACQ, Agoraphobic Cognitions Questionnaire, scales on frequency of thoughts and belief in thoughts; BSQ, Body Sensations Questionnaire.*

The results of all outcome measures at 12 months FU are presented in **Table 4**. No parameters showed significant *F*-values, although all parameters tended toward more improvement in the CBT group, with the exception of the symptoms of depression measured by the BDI. All ESs were in the range of medium (0.50–0.80) or high (above 0.80) (Cohen, 1988). On four of the 10 parameters measured, the results from the CBT treatment condition were in a higher ES group than the TFT.

**Table 4:** Means and standard deviations for patients treated with CBT (*n* = 36) or TFT (*n* = 36), measured: (1) immediately before treatment; (2) after treatment; (3) 12 months after treatment.

	(1) Immediately before treatment		(2) Immediately after treatment			(3) Twelve months after treatment				
					
	*M*	*SD*	*M*	*SD*	*d*	*M*	*SD*	*d*	*F*(1,72)	*P*
**ADIS anxiety**										
CBT	3.02	1.23	1.36	1.41	1.32	1.66	1.67	0.93
TFT	3.48	1.38	1.99	1.76	1.03	1.93	1.60	1.06	0.68	0.51
**ADIS avoidance**										
CBT	2.84	1.26	1.15	1.41	1.33	1.45	1.71	0.92
TFT	3.25	1.51	1.74	1.80	1.06	1.67	1.60	1.10	0.76	0.47
**ADIS inference**										
CBT	5.92	1.59	2.58	2.30	1.40	2.53	2.93	1.25
TFT	5.69	1.80	3.44	2.59	0.99	3.14	2.60	1.04	2.00	0.14
**ADIS distress**										
CBT	5.89	1.64	2.50	2.36	1.48	3.00	2.81	1.12
TFT	6.17	1.65	3.72	2.59	1.00	3.42	2.57	1.09	1.58	0.21
**MI alone**										
CBT	3.31	0.56	2.23	0.81	1.49	2.24	0.89	1.33
TFT	3.49	0.67	2.58	1.05	0.89	2.51	1.12	0.87	0.36	0.70
**BDI**										
CBT	14.6	9.37	10.1	9.16	0.78	10.8	8.41	0.55
TFT	15.0	11.1	9.81	10.2	0.59	10.7	11.1	0.53	0.07	0.93
**BAI**										
CBT	25.0	12.4	13.2	13.0	1.02	13.6	12.2	0.88
TFT	23.1	13.7	15.4	14.2	0.54	14.2	14.5	0.62	0.95	0.39
**ACQ freq.**										
CBT	2.11	0.55	1.75	0.60	0.73	1.59	0.51	0.85
TFT	2.28	0.65	1.80	0.71	0.73	1.81	0.61	0.70	0.91	0.41
**ACQ belief**										
CBT	25.9	14.3	14.1	13.2	0.78	9.75	9.23	0.95
TFT	28.1	13.8	17.3	18.7	0.64	18.4	17.2	0.59	1.82	0.17
**BSQ**										
CBT	2.66	0.73	2.06	0.70	0.78	1.91	0.64	0.95
TFT	2.70	0.89	2.09	0.97	0.67	2.20	0.86	0.50	1.32	0.27


*Effect sizes (Cohen’s *d*) were estimated in relation to before-treatment values. F values were computed as a Condition X Time effect across the assessments.*

*CBT, cognitive behavioral therapy; TFT, thought field therapy; ADIS anxiety, mean symptom score on anxiety; ADIS avoidance, mean symptom score on avoidance; ADIS inference, inference on daily life from agoraphobic symptoms; ADIS distress, distress in daily life from agoraphobic symptoms; MI alone, Mobility Inventory, subscale alone; BDI, Beck Depression inventory; BAI, Beck Anxiety Inventory; ACQ, Agoraphobic Cognitions Questionnaire, scales on frequency of thoughts and belief in thoughts; BSQ, Body Sensations Questionnaire.*

At the 12-month FU, 18 (50%) of the CBT patients and 10 (28%) of the TFT patients no longer met the diagnostic criteria for agoraphobia (*P* = 0.09).

Nine (25%) TFT patients had one or more Axis II diagnoses at the start of the study, while at the 12-month FU six no longer had an Axis II diagnosis. Among the CBT patients, five patients (14%) had an Axis II diagnosis at the start of the study, whereas two of these had no Axis II diagnosis at the 12-month FU.

Six (18%) CBT patients and one TFT patient reported side effects. Among the CBT patients, three reported increased intensity of symptoms, and the other three that the treatment was more unpleasant or tiresome than expected. One TFT patient re-experienced traumatic memories that were not successfully treated during the five TFT sessions, and she continued to remember such memories after the end of treatment, therefore receiving additional treatment immediately after the FU period.

Sixty-three (91%) of the 69 patients who completed the study were asked if they had negative experiences during the research project, but none of the CBT patients reported this. One TFT patient was disappointed at only receiving five sessions, despite an approximate 50% symptom reduction from pre to post treatment.

In 66 of the 69 patients who met to FU (CBT = 33/34 and TFT = 33/35) the ADIS interviewers were instructed to assess at the end of the interview if any information from the patient on the type of therapy received was present (yes/no), and were asked to guess which therapy the patient received if they had some idea. For seven of the patients (CBT = 3, TFT = 4) the interviewers got information about type of therapy, and thus making a guess was not relevant. For 44 patients (CBT = 23, TFT = 21) the interviewers stated that they had no information, but still did not make any guess of type of therapy the patients had received. For 15 patients (CBT = 7, TFT = 8) the interviewers stated that they had no information and made a guess. They were correct in 11 patients (CBT *n* = 5, 71%, TFT *n* = 6, 75%).

## Discussion

This randomized, controlled trial showed that TFT did better than the WL. TFT did not demonstrate results different from CBT, particularly not on the two primary effect variables, which were scored by raters blinded for treatment conditions. In their study from Barlow et al. (2000) found that CBT was superior to a placebo in panic disorders. Although the WLC is not equivalent to the placebo, our study is in agreement with their results (**Figure 2**), also taking into consideration the above-mentioned results for CBT, in addition to a study by Loerch et al. (1999), which demonstrated that CBT was superior to the placebo. The fact that TFT in our study showed results comparable to CBT is promising for TFT, but not conclusive.

The results of the present study are consistent with those found by Wells et al. (2003) in their study of EFT for small animal phobia. Our findings are also consistent with those of the other five RCTs on clinical populations with an anxiety disorder, including two on TFT (Schoninger and Hartung, 2010; Irgens et al., 2012) and three on EFT (Baker and Siegel, 2010; Jones et al., 2011; Salas et al., 2011), as described in the review by Feinstein (2012). While the TFT in the present study demonstrates positive results as a treatment for agoraphobia, Goldstein et al. (2000) showed poor results for EMDR in treating panic disorder with agoraphobia.

This is the first study reporting on the effectiveness of TFT applied for patients with agoraphobia. Because CBT is recommended in the NICE guidelines, its use as an active control is justified (Pilling et al., 2011). Pre-post, Norton and Price (2007) reported an average weighted ES for cognitive therapy for panic disorder with agoraphobia to be 1.37. This fits in with our finding of a Cohen’s *d* of 1.31 for anxiety and 1.32 for avoidance on the ADIS sum score, pre-post, for the CBT group.

However, we cannot be sure that TFT-mediated factors have led to beneficial changes; these changes may partly be caused by known specific factors such as exposure. They may also partly be caused by non-specific therapeutic factors such as hope, expectancy and alliance. Still, it is not likely that all the beneficial effects are due to these factors. Recently, Gloster et al. (2011) performed a study where the changes in MI scores from pre to post treatment and at 6 months FU provide results comparable with those found in our study. The patients in the Gloster et al. (2011) study received a 12-session CBT treatment with special focus on different exposure regimens. We find it unlikely that five sessions of TFT, with the only exposure being that the patient is thinking about their anxiety and the situation precipitating it, should produce a similar effect as that reported in the study by Gloster et al. (2011) if the effect was due only to exposure and non-specific therapeutic factors. However, the importance of the effects of non-specific or common factors is difficult to assess due to a lack of randomized studies on such common factors in psychotherapy (Crits-Christoph et al., 2014).

### Strengths and Limitations

A major strength of this study is that we have compared the TFT to a well-established therapy for agoraphobia, using a scientifically sound methodology. We have maintained clinical relevance by applying wide inclusion criteria, with the use of a minimum inclusion score of 2.5 on the MI-AAL ensuring that all patients enrolled in the study had serious symptoms and were in need of treatment for agoraphobia. It is a strength that only a few patients dropped out of therapy, and that only three were lost to FU. Another strength is that aside from the TFT therapist, the rest of the study personnel, as well as the authors worked in the ordinary psychiatric mental health care system. Five of the seven authors who are clinicians, are teachers and supervisors in CBT, including the main author, who also conducted the study. Finally, a major strength of this study is its high external validity. It has followed commonly accepted and widely used regimens for both the CBT and the TFT conditions, in a clinically representative sample, and is therefore close to how these therapies are practiced in daily clinical work.

It is a limitation that 8 patients used BZ regularly and 14 occasionally, **Table 2**. BZ may reduce the effect of CBT, as their use can represent a safety behavior for the patient (Bennett-Levy et al., 2004) and it may dampen feared sensations and thus limit the possibility of exposure and testing. Although this has not been studied for TFT, since imaginal exposure is a major part of this therapy it is reason to believe that BZ may reduce the effect of TFT as for CBT. To ascertain a high external validity we still chose to include patients using BZ, because not all patients in clinical practice end their use of these medicines even if asked to do so. We found a negative effect of benzodiazepine use in our study upon the primary effect variable, but we found no differences between CBT and TFT, suggesting that such use did not influence the relative effect of the two treatments.

One limitation is the lack of a placebo condition. In a study from Waite and Holder (2003), EFT demonstrated a significant decrease in self-reported symptoms compared to a control group that did not receive any treatment, but a placebo group tapping other points exhibited a symptom decrease similar to that of the EFT group. We consider that a proper placebo condition could not be established, because it would be impossible to ensure that both therapists and patients would be blinded to giving or receiving correct or placebo tapping, as the TFT procedure was readily available on the internet. As CBT has shown to be better than placebo (Loerch et al., 1999; Barlow et al., 2000) we found it acceptable to use a WLC in our study instead of placebo.

A possible limitation of our study is that the diagnostic interviews at FU that applied the MINI PLUS and the SCID II were performed by the principal investigator, who was not blinded to treatment conditions, though great care was done to avoid biases. Therefore, meeting the diagnostic criteria for agoraphobia at FU was not used as an outcome measure. Nonetheless, we still chose to report these findings since they favor the conservative hypothesis of CBT being the better treatment. It is a weakness that 47 of the 227 ADIS interviews were performed by the principal investigator. The reasons for this were practical and unavoidable.

The fact that 21 patients contacted the primary investigator directly without a referral, may have introduced a selection bias. However, as there were no difference between the CBT and TFT patients in number of patients taking contact directly, and there were no correlations between type of recruitment and treatment outcome, it is likely that the type of recruitment did not influence the results between CBT and TFT.

The trend on most of the secondary outcome measures differed from the results of the primary outcome measures in favor of CBT. This may be due to the fact that the TFT in this study was specifically directed toward anxiety and avoidance symptoms, whereas CBT more broadly addressed how the patients handled their thoughts and fears.

The two CBT therapists in this study were both highly experienced and extensively educated, and received a thorough supervision of their therapeutic performance by experts in CBT for agoraphobia, both before and during the treatment. It is a serious limitation of the study parameters that the TFT therapist did not meet the same standards of qualification and supervision. Still, this limitation tends to favor CBT.

Because the TFT patients in this study only received five treatment sessions, while the CBT patients got 12, the amount of therapy may have influenced the results. In order for the intervention to have clinical validity, we chose to apply the Norwegian standard TFT treatment package, which consists of five sessions (Irgens et al., 2012). Clark’s treatment manual for agoraphobia prescribes 12 sessions (Hawton et al., 1989). Again, this difference in the number of sessions favors CBT. We applied a competence scale for CBT (CTS), it is a limitation that for TFT no such competence scale exists.

The lack of broadly accepted theoretical foundations is a problem with TFT, since it is possible that other shared therapeutic factors at least partly convey the therapeutic results, such as the imagery exposure element that the TFT has in common with many other therapies (Clark and Fairburn, 1997). This has been pointed out by several critics of TFT and EFT, as McNally (2001), Herbert and Gaudiano (2001) and Pignotti and Thyer (2009). It could be argued that due to this lack of a viable theory, such a study as ours should not be performed. We had four reasons for still doing so. The first was that the first author had observed several treatment sessions with gross positive changes within a short period of time, for various anxiety conditions accompanied by considerable amounts of symptoms and decreased level of function. The second was that as a consequence of those observations the first author performed a clinical study on TFT with promising but not conclusive results (Irgens et al., 2012), and considered that it was a need for further studies to debunk or confirm these findings. The third reason was that TFT was and still is applied by many therapists (Schwarz, personal communication, December 23, 2013), so that regardless of its theoretical foundations, it is a need to study its effects. Fourth, TFT has recently become registered as an efficacious treatment for PTSD in the NREPP (National Registry of Evidence-based Programs and Practices, 2016) conducted by the SAMHSA, an agency within the U.S. Department of Health and Human services (SAMHSA).

The test for blindness demonstrated that the interviewers could identify some of the patients who had received CBT or TFT. This is a limitation for the independence of the interviewers, who had no part in the study other than performing the ADIS interviews, and who did not have any connection to alternative types of treatment, so that if present, any bias should be in favor of CBT.

## Conclusion

The study indicated that TFT may be a better than expected treatment for agoraphobia, and could be an alternative to CBT. TFT may be more time-efficient than CBT, and may be administered by a wider range of therapists. However, the study suggested that patients treated with CBT experienced a broader spectrum of beneficial effects. There is a need for comparing TFT with imaginary exposure alone to decide whether the acupressure component produces additional effects.

## Trial Registration

Clinicaltrials.gov Registry #: NCT00932919. URL: https://register.clinicaltrials.gov/prs/app/action/SelectProtocol?sid=S0000R67&selectaction=Edit&uid=U000040Y&ts=4&cx=9f6rd8.

## Author Contributions

AI conceived and conducted the study, participated in designing the study and in the data analysis and interpretation, and was the primary author for the original drafts and revisions of the manuscript. AH participated in designing the study, supervised one of the CT therapists (NB), instructed and helped with the data analyses and interpretation and has authored numerous revisions of the manuscript. TN contributed to revisions of the manuscript. VH did the blinded interviews with the ADIS on half of the patients, and helped with the statistics and revisions of the manuscript. F-MB supervised one of the CT therapists (MS), and helped with revisions of the manuscript. AP helped with the statistics and data interpretation, and contributed to revisions of the manuscript. EM participated in designing the study, and contributed to revisions of the manuscript. TD participated in designing the study, reassessed some of the diagnostic interviews and contributed to revisions of the manuscript. All the authors read and approved the final manuscript.

## Conflict of Interest Statement

The authors declare that the research was conducted in the absence of any commercial or financial relationships that could be construed as a potential conflict of interest.

## Abbreviations

ACQ: Agoraphobic Cognitions Questionnaire
ADIS: Anxiety Disorders Interview Schedule
ANCOVA: analyses of covariance
ANOVA: analyses of variance
BAI: Beck Anxiety Inventory
BDI: Beck Depression Inventory
BSQ: Body Sensations Questionnaire
BZ: benzodiazepines
CBT: cognitive behavior therapy
CI: confidence interval
CTS: Cognitive Therapy Scale
DSM-IV: diagnostic and statistical manual of mental disorders
EFT: emotional freedom techniques
EMDR: eye movement desensitization and reprocessing
ES: effect size
EXTRA: Norwegian Extra Foundation for Health and Rehabilitation
FU: follow-up
ICD-10: international statistical classification of diseases and health related problems version 10
LOCF: last observation carried forward
MI: Mobility Inventory
MI-AAL: Mobility Inventory Alone
MINI PLUS: Mini Plus International Neuropsychiatric Interview
NREPP: National Registry of Evidence-based Programs and Practices
PDA: panic disorder with agoraphobia
PTSD: post-traumatic stress disorder
RCT: randomized controlled trial
SAMHSA: Substance Abuse and Mental Health Services Administration
SCID II: Structured Clinical Interview for DSM Personality Disorders
SPSS: statistical package for the social sciences
SUDs: subjective units of discomfort
TFT: thought field therapy
TIR: traumatic incident reduction
WL: wait-list

## References

[B1] American Psychiatric Association (1994). *Diagnostic and Statistical Manual of Mental Disorders: DSM-IV*. Washington, DC: American Psychiatric Association.

[B2] Andrade J. Feinstein D. (ed.) (2003). “Preliminary report of the first large scale study of energy psychology,” in *Energy Psychology Interactive: An Integrated Book and CD Program for Learning the Fundamentals of Energy Psychology*, ed. Feistein D. (Ashland, OR: Norton Professional Books).

[B3] Arntz A. Tiesema M. Kindt M. (2007). Treatment of PTSD: a comparison of imaginal exposure with and without imagery rescripting. *J. Behav. Ther. Exp. Psychiatry* 38 345–370. 10.1016/j.jbtep.2007.10.006 18005935

[B4] Baker A. H. Siegel L. (2010). Emotional Freedom Techniques (EFT) reduces intense fears: a partial replication and extension of Wells, Polglase, Andrews, Carrington, & Baker (2003). *Energy Psychol.* 2 13–29. 10.9769/EPJ.2010.2.2.AHB.LSS

[B5] Barlow D. H. Gorman J. M. Shear M. Woods S. W. (2000). Cognitive-behavioral therapy, imipramine, or their combination for panic disorder: a randomized controlled trial. *JAMA* 283 2529–2536. 10.1001/jama.283.19.2529 10815116

[B6] Beck A. Epstein N. Brown G. Steer R. A. (1988). An inventory for measuring clinical anxiety: psychometric properties. *J. Consult. Clin. Psychol.* 56 893–897. 10.1037/0022-006X.56.6.893 3204199

[B7] Beck A. Ward C. Mendelson M. Mock J. Erbaugh J. (1961). An inventory for measuring depression. *Arch. Gen. Psychiatry* 4 561–571. 10.1001/archpsyc.1961.01710120031004 13688369

[B8] Beck A. T. (1979). *Cognitive Therapy of Depression.* New York, NY: Guilford press.

[B9] Bennett-Levy J. E. Butler G. E. Fennell M. E. Hackman A. E. Mueller M. E. Westbrook D. E. (2004). *Oxford Guide to Behavioural Experiments in Cognitive Therapy.* Oxford: Oxford University Press 10.1093/med:psych/9780198529163.001.0001

[B10] Bergmann U. (2010). EMDR’s neurobiological mechanisms of action: a survey of 20 years of searching. *J. EMDR Pract. Res.* 4 22–42. 10.1891/1933-3196.4.1.22

[B11] Brown T. A. Di Nardo P. A. Lehman C. L. Campbell L. A. (2001). Reliability of DSM-IV anxiety and mood disorders: implications for the classification of emotional disorders. *J. Abnorm. Psychol.* 110 49–58. 10.1037/0021-843X.110.1.49 11261399

[B12] Butler A. C. Chapman J. E. Forman E. M. Beck A. T. (2006). The empirical status of cognitive-behavioral therapy: a review of meta-analyses. *Clin. Psychol. Rev.* 26 17–31. 10.1016/j.cpr.2005.07.003 16199119

[B13] Callahan R. J. Trubo R. (2001). *Tapping the Healer within: Using thought Field Therapy to Instantly Conquer your Fears, Anxieties, and Emotional Distress.* Chicago, IL: Contemporary Books.

[B14] Chambless D. L. Caputo G. Bright P. Gallagher R. (1984). Assessment of fear of fear in agoraphobics: the body sensations questionnaire and the agoraphobic cognitions questionnaire. *J. Consult. Clin. Psychol.* 52 1090–1097. 10.1037/0022-006X.52.6.1090 6520279

[B15] Chambless D. L. Caputo G. Jasin S. E. Gracely E. J. Williams C. (1985). The mobility inventory for agoraphobia. *Behav. Res. Ther.* 23 35–44. 10.1016/0005-7967(85)90140-8 3985915

[B16] Church D. (2014). Reductions in pain, depression, and anxiety symptoms after PTSD remediation in veterans. *Explore* 10 162–169. 10.1016/j.explore.2014.02.005 24767263

[B17] Church D. Brooks A. J. (2014). CAM and energy psychology techniques remediate PTSD symptoms in veterans and spouses. *Explore* 10 24–33. 10.1016/j.explore.2013.10.006 24439093

[B18] Church D. Feinstein D. (2013). “Energy psychology in the treatment of PTSD: psychobiology and clinical principles,” in *Psychology of Trauma*, ed. Van Leeuwen T. (Hauppauge, NY: Nova Science Publishers), 211–224.

[B19] Church D. Palmer-Hoffman J. (2014). TBI symptoms improve after PTSD remediation with emotional freedom techniques. *Traumatology* 20 172–181. 10.1037/h0099831

[B20] Church D. Pina O. Reategui C. Brooks A. (2012). Single-session reduction of the intensity of traumatic memories in abused adolescents after EFT: a randomized controlled pilot study. *Traumatology* 18 73–79. 10.1177/1534765611426788

[B21] Clark D. M. Fairburn C. G. (1997). *Science and Practice of Cognitive Behaviour Therapy.* Oxford: Oxford University Press.

[B22] Cohen J. (1988). *Statistical Power for the Behavioral Sciences.* Hillsdale, NJ: Lawrence Erbaum Associates.

[B23] Collins P. Y. Patel V. Joestl S. S. March D. Insel T. R. Daar A. S. (2011). Grand challenges in global mental health. *Nature* 475 27–30. 10.1038/475027a 21734685 PMC3173804

[B24] Craig G. (2007). *The EFT Manual (Emotional Freedom Techniques).* Available at: http://www.emofree.com/ [accessed February 23, 2015].

[B25] Craske M. G. DeCola J. P. Sachs A. D. Pontillo D. C. (2003). Panic control treatment for agoraphobia. *J. Anxiety Disord.* 17 321–333. 10.1016/S0887-6185(02)00203-7 12727125

[B26] Crits-Christoph P. Chambless D. L. Markell H. M. (2014). Moving evidence-based practice forward successfully: commentary on Laska, Gurman, and Wampold. *Psychotherapy* 51 491–495. 10.1037/a0036508 25419728

[B27] DiNardo P. A. Brow T. A. Barlow D. H. (1994). *Anxiety Disorders Interview Schedule for DSM-IV: Life Time Version: Client Interview Schedule.* Oxford: Oxford University Press.

[B28] Eia A. T. (2012). *Tankefeltterapi: Gjør det selv: Fobier.* Grimstad: Trivsel i hverdagen.

[B29] Feinstein D. (2010). Rapid treatment of PTSD: why psychological exposure with acupoint tapping may be effective. *Psychotherapy* 47 385–402. 10.1037/a0021171 22402094

[B30] Feinstein D. (2012). Acupoint stimulation in treating psychological disorders: evidence of efficacy. *Rev. Gen. Psychol.* 16 364–380. 10.1037/a0028602

[B31] Foa E. B. Hembree E. A. Rothbaum B. O. (2007). *Prolonged Exposure Therapy for PTSD: Emotional Processing of Traumatic Experiences: Therapist Guide.* New York, NY: Oxford University Press 10.1093/med:psych/9780195308501.001.0001

[B32] Foa E. B. Kozak M. J. (1986). Emotional processing of fear: exposure to corrective information. *Psychol. Bull.* 99 20–35. 10.1037/0033-2909.99.1.20 2871574

[B33] Gloster A. T. Wittchen H.-U. Einsle F. Lang T. Helbig-Lang S. Fydrich T. (2011). Psychological treatment for panic disorder with agoraphobia: a randomized controlled trial to examine the role of therapist-guided exposure in situ in CBT. *J. Consult. Clin. Psychol.* 79 406–420. 10.1037/a0023584 21534651

[B34] Goldstein A. J. de Beurs E. Chambless D. L. Wilson K. A. (2000). EMDR for panic disorder with agoraphobia: comparison with waiting list and credible attention-placebo control conditions. *J. Consult. Clin. Psychol.* 68 947–956. 10.1037/0022-006X.68.6.947 11142547

[B35] Grant B. F. Hasin D. S. Stinson F. S. Dawson D. A. Goldstein R. B. Smith S. (2006). The epidemiology of DSM-IV panic disorder and agoraphobia in the United States: results from the National Epidemiologic Survey on Alcohol and Related Conditions. *J. Clin. Psychiatry* 67 363–374. 10.4088/JCP.v67n0305 16649821

[B36] Hartung J. Galvin M. (2003). *Energy Psychology and EMDR: Combining Forces to Optimize Treatment.* New York, NY: WW Norton.

[B37] Hawton K. Salkovskis P. M. Kirk J. Clark D. M. (1989). *Cognitive Behaviour Therapy for Psychiatric Problems: A Practical Guide.* New York, NY: Oxford University Press 10.1093/med:psych/9780192615879.001.0001

[B38] Herbert J. D. Gaudiano B. A. (2001). The search for the holy grail: heart rate variability and thought field therapy. *J. Clin. Psychol.* 57 1207–1214. 10.1002/jclp.1087 11526607

[B39] Holmaas O. E. (2017). Available at: http://www.tftcoaching.no [accessed May 07, 2017].

[B40] Hui K. K. S. Liu J. Makris N. Gollub R. L. Chen A. J. W. I Moore C. (2000). Acupuncture modulates the limbic system and subcortical gray structures of the human brain: evidence from fMRI studies in normal subjects. *Hum. Brain Mapp.* 9 13–25. 10.1002/(SICI)1097-0193(2000)9:1<13::AID-HBM2>3.0.CO;2-F 10643726 PMC6871878

[B41] Irgens A. Dammen T. Nysæter T. E. Hoffart A. (2012). Thought Field Therapy (TFT) as a treatment for anxiety symptoms: a randomized controlled trial. *Explore* 8 331–338. 10.1016/j.explore.2012.08.002 23141789

[B42] Jones S. Thornton J. Andrews H. (2011). Efficacy of Emotional Freedom Techniques (EFT) in reducing public speaking anxiety: a randomized controlled trial. *Energy Psychology* 3 19–32. 10.9769/EPJ.2011.3.1.SJJ.JAT.HBA

[B43] Kringlen E. Torgersen S. Cramer V. (2001). A Norwegian psychiatric epidemiological study. *Am. J. Psychiatry* 158 1091–1098. 10.1176/appi.ajp.158.7.1091 11431231

[B44] Loerch B. Graf-Morgenstern M. Hautzinger M. Schlegel S. Hain C. Sandmann J. (1999). Randomised placebo-controlled trial of moclobemide, cognitive-behavioural therapy and their combination in panic disorder with agoraphobia. *Br. J. Psychiatry* 174 205–212. 10.1192/bjp.174.3.205 10448444

[B45] McNally R. J. (2001). Tertullian’s motto and Callahan’s method. *J. Clin. Psychol.* 57 1171–1174. 10.1002/jclp.1083 11526603

[B46] National Guideline Clearing House (2011). *Generalised Anxiety Disorder and Panic Disorder (With or Without Agoraphobia) in Adults. Management in Primary, Secondary and Community Care.* Rockville, MD: Agency for Healthcare Research and Quality (AHRQ).

[B47] National Registry of Evidence-based Programs and Practices (2016). *National Registry of Evidence-based Programs and Practices.* Available at: http://www.nrepp.samhsa.gov [accessed May 29, 2016].

[B48] Norton P. J. Price E. C. (2007). A meta-analytic review of adult cognitive-behavioral treatment outcome across the anxiety disorders. *J. Nerv. Ment. Dis.* 195 521–531. 10.1097/01.nmd.0000253843.70149.9a 17568301

[B49] Pignotti M. Thyer B. (2009). Some comments on “Energy psychology: a review of the evidence”: premature conclusions based on incomplete evidence? *Psychotherapy* 46 257–261. 10.1037/a0016027 22122623

[B50] Pilling S. Whittington C. Taylor C. Kendrick T. (2011). Identification and care pathways for common mental health disorders: summary of NICE guidance. *BMJ* 342:d2868 10.1136/bmj.d2868 21610049

[B51] Ruden R. A. (2007). A model for disrupting an encoded traumatic memory. *Traumatology* 13 71–75. 10.1177/1534765607299909

[B52] Salas M. M. Brooks A. J. Rowe J. E. (2011). The immediate effect of a brief energy psychology intervention (emotional freedom techniques) on specific phobias: a pilot study. *Explore* 7 155–161. 10.1016/j.explore.2011.02.005 21571234

[B53] Schoninger B. Hartung J. (2010). Changes on self-report measures of public speaking anxiety following treatment with Thought Field Therapy. *Energy Psychol.* 2 13–26. 10.9769/EPJ.2010.2.1.BS.JH

[B54] Sheehan D. V. Lecrubier Y. Sheehan K. Amorim P. Janavs J. Weiller E. (1998). The Mini-International Neuropsychiatric Interview (M.I.N.I): the development and validation of a structured diagnostic psychiatric interview for DSM-IV and ICD-10. *J. Clin. Psychiatry* 59(Suppl. 20), 22–33. 9881538

[B55] Uldal M. (2007). *De sa det Ikke var Mulig.* Oslo: Flux forlag.

[B56] Waite W. L. Holder M. D. (2003). Assessment of the emotional freedom technique: an alternative treatment for fear. *Sci. Rev. Ment. Health Pract.* 2 20–26.

[B57] Wells S. Polglase K. Andrews H. B. Carrington P. Baker A. H. (2003). Evaluation of a meridian-based intervention, Emotional Freedom Techniques (EFT), for reducing specific phobias of small animals. *J. Clin. Psychol.* 59 943–966. 10.1002/jclp.10189 12945061

[B58] Westra H. A. Constantino M. J. Arkowitz H. Dozois D. J. (2011). Therapist differences in cognitive-behavioral psychotherapy for generalized anxiety disorder: a pilot study. *Psychotherapy* 48 283–292. 10.1037/a0022011 21688930

[B59] Wolpe J. (1990). *The Practice of Behavior Therapy*, 4th Edn Elmsford, NY: Pergamon Press.

